# Shared Control of Bimanual Robotic Limbs With a Brain-Machine Interface for Self-Feeding

**DOI:** 10.3389/fnbot.2022.918001

**Published:** 2022-06-28

**Authors:** David A. Handelman, Luke E. Osborn, Tessy M. Thomas, Andrew R. Badger, Margaret Thompson, Robert W. Nickl, Manuel A. Anaya, Jared M. Wormley, Gabriela L. Cantarero, David McMullen, Nathan E. Crone, Brock Wester, Pablo A. Celnik, Matthew S. Fifer, Francesco V. Tenore

**Affiliations:** ^1^Department of Research and Exploratory Development, Johns Hopkins University Applied Physics Laboratory, Laurel, MD, United States; ^2^Department of Biomedical Engineering, Johns Hopkins University School of Medicine, Baltimore, MD, United States; ^3^Department of Physical Medicine and Rehabilition, Johns Hopkins Medicine, Baltimore, MD, United States; ^4^National Institute of Mental Health, National Institutes of Health, Bethesda, MD, United States; ^5^Department of Neurology, Johns Hopkins Medicine, Baltimore, MD, United States

**Keywords:** human machine teaming, brain computer interface (BCI), bimanual control, robotic shared control, activities of daily living (ADL)

## Abstract

Advances in intelligent robotic systems and brain-machine interfaces (BMI) have helped restore functionality and independence to individuals living with sensorimotor deficits; however, tasks requiring bimanual coordination and fine manipulation continue to remain unsolved given the technical complexity of controlling multiple degrees of freedom (DOF) across multiple limbs in a coordinated way through a user input. To address this challenge, we implemented a collaborative shared control strategy to manipulate and coordinate two Modular Prosthetic Limbs (MPL) for performing a bimanual self-feeding task. A human participant with microelectrode arrays in sensorimotor brain regions provided commands to both MPLs to perform the self-feeding task, which included bimanual cutting. Motor commands were decoded from bilateral neural signals to control up to two DOFs on each MPL at a time. The shared control strategy enabled the participant to map his four-DOF control inputs, two per hand, to as many as 12 DOFs for specifying robot end effector position and orientation. Using neurally-driven shared control, the participant successfully and simultaneously controlled movements of both robotic limbs to cut and eat food in a complex bimanual self-feeding task. This demonstration of bimanual robotic system control *via* a BMI in collaboration with intelligent robot behavior has major implications for restoring complex movement behaviors for those living with sensorimotor deficits.

## Introduction

Individuals living with sensorimotor impairments, such as a spinal cord injury, are faced with challenges in navigating their environments and completing daily tasks, including self-feeding. Brain-machine interfaces (BMI) have the potential to increase the independence of such individuals by providing control signals to prosthetic limbs and re-enabling activities of daily living (ADLs) (Collinger et al., 2013; Aflalo et al., 2015; Thomas et al., 2020).

Cortical BMIs can be used to restore function by decoding neural signals for a variety of applications including handwriting (Willett et al., 2021), restoring speech (Moses et al., 2021), perceiving artificial stimulation (Armenta Salas et al., 2018; Fifer et al., 2022), controlling external robotic limbs with closed-loop sensory feedback (Flesher et al., 2021), or combined with other assistive technologies (McMullen et al., 2014; Downey et al., 2016). Others have demonstrated the use of cortical BMIs for driving functional electrical stimulation to allow users to volitionally control their limb for completing tasks such as drinking (Ajiboye et al., 2017; Colachis et al., 2018).

As invasive cortical BMIs become more advanced, one challenge that remains is robust control of bimanual robotic limbs with high degrees of freedom (DOFs). Researchers have demonstrated bimanual control of virtual arms using neural signals from bilateral frontal and parietal cortical areas in non-human primates (Ifft et al., 2013). Prior work has demonstrated effective control of seven (Collinger et al., 2013) and even up to 10 (Wodlinger et al., 2015) DOFs in individuals using an invasive cortical BMI to move anthropomorphic robotic limbs; however, despite these impressive advances the need for bimanual control of two robotic limbs for more complex tasks of daily living requires control over as many as 34 DOFs, if using highly dexterous robotic limbs (Johannes et al., 2020). To address this challenge, advanced strategies, such as shared control, could help significantly reduce the required DOFs needed to effectively complete tasks requiring two arms while using a BMI.

Shared control systems, where the BMI user and a semi-autonomous robot combine efforts to accomplish tasks, can further increase user independence in a variety of ways. One method is a form of *supervisory shared control* where the robot knows how to perform tasks and the BMI user provides target goals for the robot (Katyal et al., 2013; McMullen et al., 2014; Tang and Zhou, 2017). In this case, an “inner loop” control system drives robot movement and the BMI user provides “outer loop” control, such as telling the robot which object to pick up. The approach we describe here is a form of *collaborative shared control*, in which a subset of the robot DOFs are controllable by the BMI user at select task-specific times, or through mode-switches initiated by the user. The BMI user and robot share the main control loop and the intent is to leverage the limited command signals provided by the BMI to enable the user to customize robot behavior. A third approach could be to combine supervisory and collaborative shared control, where for a given task some subtasks are performed by the robot given target goals (supervisory—e.g., BMI user indicates which glass to pick up) whereas other subtasks involve real time input from the BMI user (collaborative—e.g., user controls how quickly to pour contents of glass).

**Figure 1:**
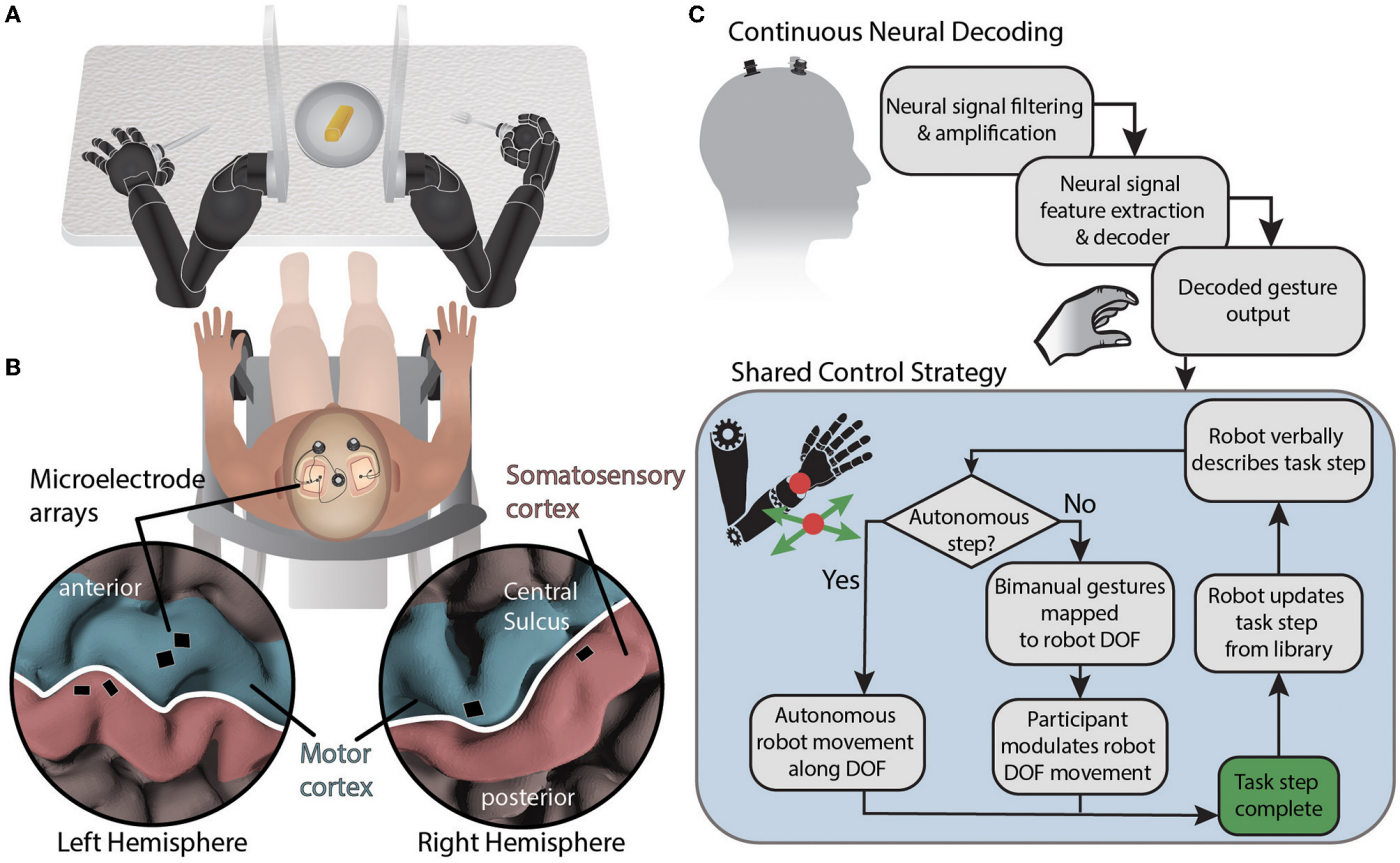
System diagram for BMI-based shared control of bimanual robotic limbs. **(A)** Movements are decoded from neural signals through the brain-machine interface and mapped to two external robotic limbs while using a collaborative shared human-machine teaming control strategy to complete a self-feeding task requiring simultaneous bimanual manipulations. **(B)** NeuroPort electrode arrays (Blackrock Neurotech) implanted in the motor and somatosensory regions of the left and right hemispheres record neural activity. **(C)** Neural data is streamed from the cortical implants and processed before being decoded. Decoded gestures are passed to the shared control strategy for mapping onto robot degrees of freedom depending on the current state of the task. Autonomous portions of the task are performed by the robot while semi-autonomous steps are controlled in part by the participant using attempted gestures to modulate a subset of robotic limb end effector degrees of freedom using the current DOF mapping. The degrees of freedom controlled *via* BMI are based on a task library accessed by the robot.

Here, we describe an experimental setup aimed at testing a collaborative shared control approach in which a BMI user controlling up to four combined bilateral degrees-of-freedom completes a bimanual self-feeding task similar to Task 1 of the Arm Motor Ability Test [AMAT (Kopp et al., 1997)] (Figure 1A).

## Methods

### Human Participant Testing and Regulatory Information

The participant recruited for this study was a 49 year old male who suffered from a C5 sensory /C6 motor spinal cord injury ≈30 years prior to this testing. The injury was categorized as a B (incomplete) on the American Spinal Cord Injury Association Impairment Scale. In particular, the participant retained some movement in shoulder and elbow, partial movement of wrist (extension only), and minimal to no movement of fingers. The participant was implanted with six NeuroPort electrode arrays (Blackrock Neurotech; Salt Lake City, UT) in the motor and somatosensory cortices of both brain hemispheres. Additional details can be found in McMullen et al. (2021) and Fifer et al. (2022). Briefly, two 96 channel arrays (10 × 10 layout spanning 4 × 4 mm) were placed in the dominant (left) primary motor cortex and two 32 channel arrays (within a 6 ×10 layout spanning 4 ×2.5 mm) were placed in the somatosensory cortex. In the non-dominant (right) hemisphere, one 96 channel array and one 32 channel array were located in the primary motor and somatosensory cortices, respectively (Figure 1B). The arrays notionally covered neural regions representing both the left and right limbs as reported in previous studies (Thomas et al., 2020; Christie et al., 2021; McMullen et al., 2021; Osborn et al., 2021a,b; Fifer et al., 2022).

This study was conducted under an approved Food and Drug Administration (FDA) Investigational Device Exemption (G170010). This study was registered on ClinicalTrials.gov (NCT03161067) and was reviewed and approved by the Johns Hopkins Institutional Review Board (IRB). Surgical explantation of the investigational device occurred approximately a year after this testing.

### Neural Decoding

Neural signals were recorded from the implanted microelectrode arrays using a wired connection from the implants to three 128 channel Neuroport Neural Signal Processors (Blackrock Neurotech). Before each experiment session began, voltage thresholds for each electrode were set to −3.25 times the root-mean-square (RMS) voltage of the neural signal during a resting state. Neural spikes from the implanted arrays were recorded at 30 kHz and the firing rate for each electrode was calculated using 30 ms bins. Spiking activity in each channel, specifically the square-root of the firing rate, was normalized at each time point using a streaming z-score calculation (using the channel-specific mean and standard deviation) over the preceding 60 s (Downey et al., 2017; Colachis et al., 2018).

For this study, our team leveraged a gesture-based 2D control strategy, wherein each direction was mapped to a gesture: up (open hand), down (2-finger pinch), toward midline (wrist flex), away from midline (wrist extend), and no movement (hand rest). This mapping was familiar to the participant prior to this study. This control strategy was chosen due to limited directional tuning of spiking signals, and resulting poor performance of classical continuous direction-based velocity control. The participant performed a target-reach training task at the beginning of the experiment where they attempted gesture-based control while watching two virtual Modular Prosthetic Limbs (vMPL) (Ravitz et al., 2013; Wester et al., 2020) move to targets located on vertical and horizontal axes. The target locations were displayed for 0.5–1 s before the vMPLs began to move to the targets. vMPL movement initiation was signaled by an audio cue, which instructed the participant to start attempting the gestures corresponding to the movement direction. The participant was instructed to attempt to perform the gestures, i.e., without inhibiting movement of fingers or wrist. The gestures were attempted and held continuously until the vMPL reached its target. The gesture-direction mapping was mirrored between the two hands, and the participant was presented with 16 repetitions of each unimanual and bimanual (parallel and anti-parallel directions) movement combination (256 total training trials). Specifically, there were 16 unimanual and bimanual gesture combinations that were trained: four unimanual movements (hand open, pinch, wrist extend/flex) with the right arm and four with the left arm; two bimanual parallel movements in the horizontal directions (i.e., right wrist flex with left wrist extend and vice versa) and two bimanual anti-parallel horizontal movements (i.e., right wrist flex with left wrist flex and right wrist extend with left wrist extend); two bimanual parallel vertical movements (i.e., right open hand with left open hand and right pinch with left pinch) and two bimanual anti-parallel vertical movements (i.e., right open hand with left pinch and vice versa). Offline decoding accuracy was determined using 10-fold cross-validation. The normalized neural firing rates from each hemisphere were segmented from the training data and used to train a linear discriminant analysis (LDA) classifier for decoding intended gestures from the contralateral hand to enable simultaneous classification of bimanual movements (Thomas et al., 2020). To generate the training samples for the neural decoder, the normalized neural signals were split into 240 ms windows with a 90 ms shift. The average activity was taken for each window and labeled with the corresponding attempted hand gesture to create a training sample.

Online performance of these gesture classifiers was tested using the same target-reach task. But rather than the vMPLs being controlled by the computer, their movement direction was determined by the gestures predicted by the decoding model. Online decoding was achieved by binning the incoming neural signal and averaging the normalized firing rates from each electrode across a 240 ms buffer. A gesture prediction from the neural signal was made every 30 ms and the output was transmitted using a custom software interface to send control commands to the robot control system controlling the MPL (Johannes et al., 2020).

### Shared Control Strategy

In our shared control strategy, the participant and the bimanual manipulators are viewed as a human-robot team, and the goal is to enable the human to control a minimal set of key DOF to maximize task performance while minimizing human workload. It is a form of adjustable autonomy in which the robot nominally knows how to perform a task, and human input is used to guide and customize robot behavior (Handelman et al., 1990, 2022; National Academies of Sciences, Engineering, and Medicine, 2022).

For bimanual manipulation, the shared control system must maneuver the two MPL end effectors (i.e., the anthropomorphic robot hands) to achieve desired task goals. A total of 12 DOFs—3D position and 3D orientation (pose) of both end effectors—need to be controlled at all times by either the user or the system. Each MPL uses seven joints (three in the shoulder, one in the elbow and three in the wrist) to position and orient the end effector, and we use inverse kinematics to compute commanded joint angles for a desired end effector pose (Handelman et al., 2018).

**Figure 2:**
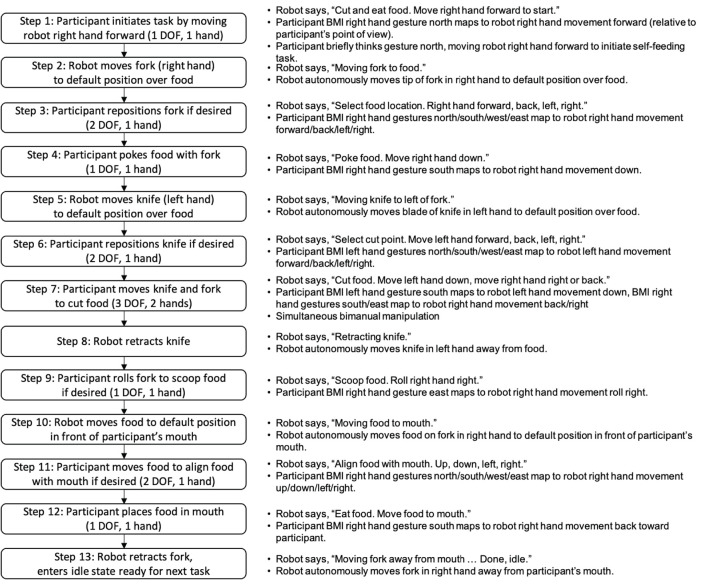
Bimanual self-feeding task flowchart. The collaborative shared control strategy enables the participant to control a minimum set of DOFs while still maximizing task performance.

In this study, the human was positioned between the robot arms and in front of a table with a plate of food (Figure 1A). The robot hands held utensils and the fingers of each robot hand remained fixed relative to the wrist. The shared control strategy was to dynamically partition the 12 controllable DOFs (position and orientation of each robot hand) into BMI-controlled and system-controlled DOF (Figure 1C). Complex tasks were manually divided into task steps. During each task step up to four DOFs were controlled by the BMI (i.e., up to two DOF on each side), and the remaining DOF were controlled by the robot control system. The active mapping of participant two-DOF input (north-south or east-west) to end effector six-DOF movement for each hand (left-right, forward-back, up-down, yaw left-right, pitch up-down, and roll left-right) was announced verbally by the robot during task execution. The overall goal was to let the robot do the majority of a task but allow the participant to take control of a subset of DOF to shape task performance to their liking. In the case of self-feeding, for example, given a plate with a variety of foods on it, we want the participant to be able to choose which food to eat, where to cut (if necessary), and the size of the piece to eat (Figure 2).

The robot end effector DOFs available to the participant were those considered most useful for the active task step. This made the shared control system easy to use, robust, and safe in its behavior, operating within a constrained task-specific envelope of motion. Sometimes the participant could control only one DOF of one end effector in one direction (“move right hand down”), sometimes two bidirectional DOFs of one end effector (“move right hand forward, back, left, right”), and sometimes three DOFs total, one associated with one effector and two associated with the other (“move left hand down, move right hand back and right”).

In this type of collaborative control strategy, where the robot is performing the majority of a multi-step task (e.g., “cut and eat food”) but the BMI user controls a subset of DOF during certain task steps, the robot must be able to estimate when the BMI user is satisfied with their input (and current robot state) and is done with the active step so that the robot can move on to the next step. For a task step where the user is providing input, the robot waits for the user to provide initial BMI input (gestures), then waits for the user to stop providing input, then moves on to the next step.

Detection of this “intentional” user activity is complicated by the nature of the BMI-generated gestures signals. Occasionally a short-lived gesture “spike” occurs that does not necessarily imply intended sustained robot arm movement by the BMI user. In order to handle this situation, we compute a *cmd_moving_raw* signal as the sum of the absolute values of the incoming active DOF, where each degree of freedom has a gesture-based value of −1, 0, or 1. This *cmd_moving_raw* signal is passed through a second-order filter (damping ratio 0.8, settling time 0.5 s) to provide *cmd_moving_filtered*. If *cmd_moving_filtered* is greater than or equal to *cmd_moving_threshold* value of 0.125 it is assumed that the BMI user is actively providing input, otherwise it is assumed the user is not providing input. This filter enables “noisy” gesture spikes to be ignored by the system while enabling sustained gestures to be interpreted as intentional activity. The robot control system monitors these values at a rate of 40 Hz, the nominal robot control system update rate.

User gestures are mapped to robot end effector velocity for the active DOF through a *velocity scaling factor*. This scaling factor needs to be tuned so that it is not too slow as to be tedious to control by the user but not so fast as to induce overshoot and oscillations. For each task step where user input is expected, the robot detects initial movement (*cmd_moving_filtered* ≥ *cmd_moving_threshold*), then looks for a lack of input activity (*cmd_moving_filtered*<*cmd_moving_threshold* for >1.1 s (*cmd_moving_wait_reset)*, at which point it considers the current step done. The robot also considers the current step done if the user BMI input switches from inactive to active at least 13 times (*cmd_moving_count_max)*. Another feature of the system is to keep the robot end effector within a bounded region (*bounding box*) relative to a task step *reference point*, such as where the robot positions the fork over the food, the knife over the food, and the food in front of the user's mouth in the self-feeding task. In some task steps the step is considered done when the edge of the bounding box is reached, such as when moving the fork down to stab food, moving the knife down to cut food, and when moving food into the user's mouth.

### Self-Feeding Task

To demonstrate shared control of bimanual robotic limbs, a decadent dessert pastry was placed on a table in front of two MPLs mounted on a stand. The participant was instructed to use neural control to guide the robotic limbs in order to cut a piece of the pastry and bring it to his mouth (Figure 1A). Consuming the pastry was optional, but the participant elected to do so given that it was delicious.

A fork and a knife were attached to the right and left MPLs, respectively. The participant controlled a subset of the position and orientation DOF of each MPL using the decoded neural signal commands and the self-feeding task was stepped through using various DOF modes to contribute to the overall feeding task as described above. Figure 2 presents the shared control self-feeding task flowchart. Trials were considered successful if the participant was able to cut the pastry using bimanual neural control and complete the self-feeding portion of the task using the MPLs.

## Results

### Neural Decoding

**Figure 3:**
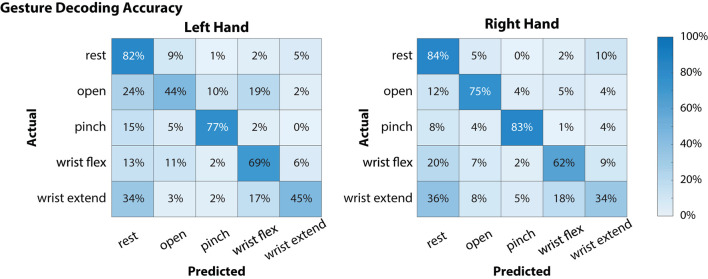
Offline decoding performance for the left and right hands during simultaneous attempted gestures (including “rest”). Contralateral neural signals were used to decode each hand (i.e., left motor and somatosensory cortex signals were used to decode right hand movements). On each hand, there were 48 instances for each of the movement classes (open, pinch, wrist flex, wrist extend) and 64 for the “rest” condition. Of the movements, “pinch” gestures were decoded with the highest accuracy for both left and right hands, while “wrist extend” was notably more difficult to decode (45 and 34% for left and right hands, respectively).

Global offline gesture decoding accuracy from the neural signals for the left and right hands was 63.5 and 67.6%, respectively (chance accuracy: 20%; see Figure 3). These offline accuracies are similar to previously reported results by our team using similar methods during simultaneous bimanual movements (Thomas et al., 2020). During online testing with the target-reach task, the participant was successful in 17 out of 20 trials (85%), indicating usability of the neural decoder for controlling the external robotic limbs in the self-feeding task.

### Bimanual Self-Feeding Task

Calibration and testing of the collaborative shared control strategy for self-feeding was performed in one session with the participant. Development of robot task behaviors and preliminary tuning of system parameters was done using simulation prior to the session.

In the current experimental setup, the robot had a priori knowledge of the approximate locations of the plate, of the food on the plate, and of the participant's mouth. Robot motion segments involving the robot moving the tip of the fork and the blade of the knife to the food, and moving the piece of food to the participant's mouth, were calibrated during testing using end effector position and orientation reference points. As noted previously, when the participant was controlling an end effector DOF, bounding box limits were placed on how far the end effector could move from the default position (reference point). This kept the robot from wandering too far from a practical pose for the active task step, helping to minimize participant effort, and kept the robot within a safe envelope relative to the participant, such as when placing food within the participant's mouth. These bounding box limits for various task steps were also calibrated during testing.

Calibration of the end effector velocity scaling factor was critical. Improper calibration would cause the robot arm to either move frustratingly slowly, requiring a great deal of participant effort (many gestures to move the robot arm a small distance), or too quickly, causing the fork or knife to overshoot the desired location. Calibration of user activity monitoring parameters *cmd_moving_threshold, cmd_moving_wait_reset*, and *cmd_moving_count_max* was also important to properly interpret when intended user activity had begun and when it had ended. Improper setting of these parameters caused task steps to end sooner than desired, before the fork or knife had been positioned as desired.

**Figure 4:**
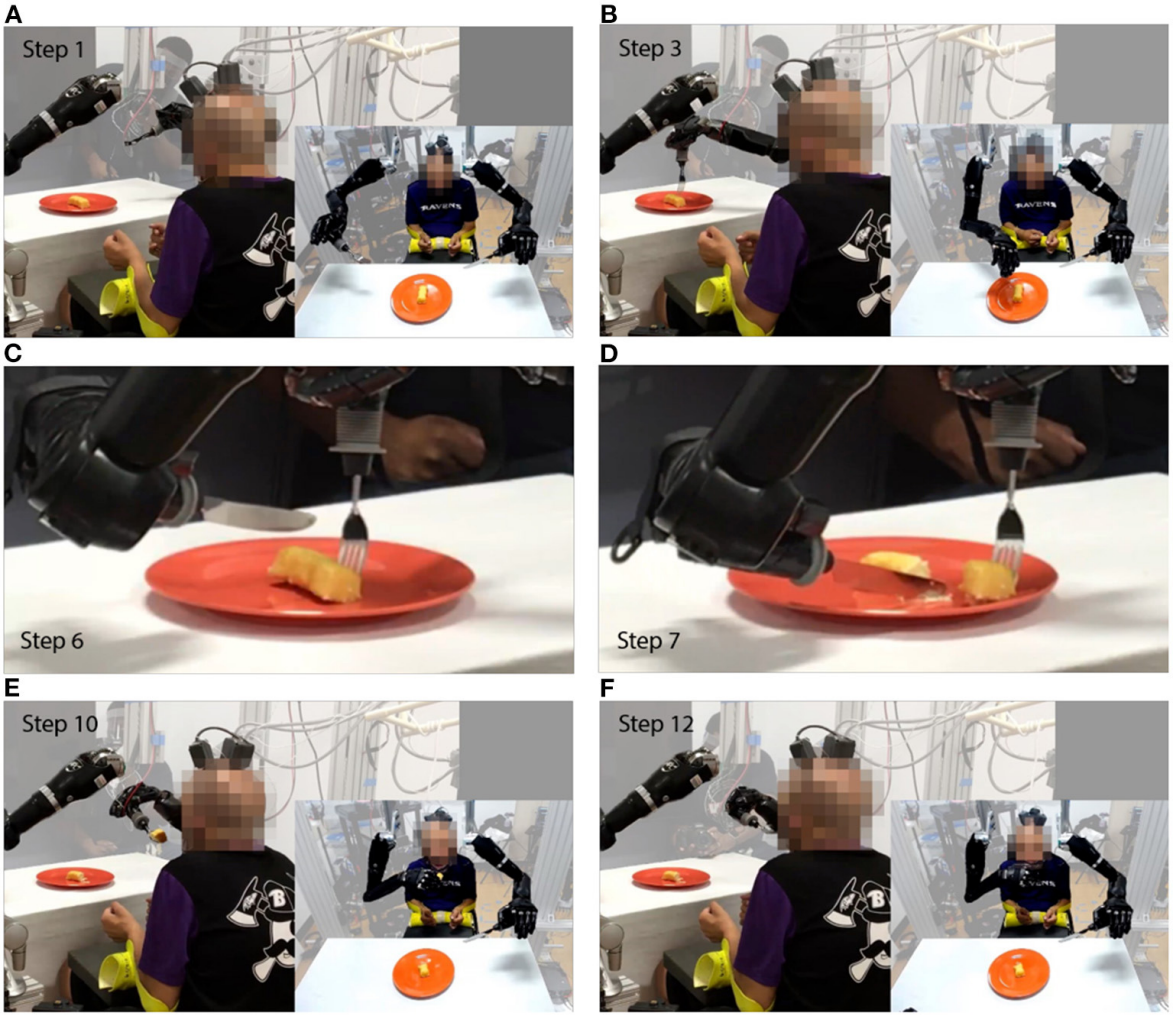
Select screenshots of self-feeding task performance. The robot holds a fork in right hand and a knife in left hand. **(A)** Step 1: Participant initiates task by moving robot right hand forward. **(B)** Step 3: Participant repositions fork horizontally to align with desired piece of food. **(C)** Step 6: Participant repositions knife horizontally to select cut point. **(D)** Step 7: Participant moves knife down and fork back and right to cut food. **(E)** Step 10: Robot moves food to default position in front of participant's mouth. **(F)** Step 12: Participant places food in mouth.

Once the shared control system was tuned, the participant could perform the self-feeding task, cutting off a reasonably sized piece of food using simultaneous bimanual commands and bringing it to his mouth without dropping it (Figure 4, Supplementary Movie 1). The test session included 37 trials, most of which involved calibration of the aforementioned parameters due to inconsistencies between the robot simulation and physical hardware. Ultimately, seven successful trials were demonstrated where the participant was able to cut off a reasonably sized piece of food and bring it to his face without dropping it using simultaneous bimanual commands (Supplementary Movie 1). While the size of the cut food was subjectively determined by the participant's preference, we note that in one of those seven trials the piece of food cut was too large to be reasonably consumed in one bite and in another one of those seven trials the cut food was much smaller. In seven partially successful trials the participant was able to cut the food but the food fell off the fork. In a total of 26 out of the 37 trials the participant performed bimanual control by employing the knife and fork in the self-feeding task, and the remaining 11 trials were halted and bimanual control was not achieved because the fork did not make contact with the food.

## Discussion

The collaborative approach to shared control presented here is intended as an exploratory proof of concept into how BMI signals might be blended with robot autonomy to enable a user to perform complex bimanual tasks that can be customized using limited BMI signals, such as which food on the plate to eat and how big a piece to cut. The ultimate goal is adjustable autonomy that leverages whatever BMI signals are available to their maximum effectiveness, enabling the human to control the few DOFs that most directly impact the qualitative performance of a task while the robot takes care of the rest. An important objective is to accommodate a wide range of BMI signal quantity and quality, both high-fidelity continuous/proportional signals and event-based/bang-bang signals such as the BMI-based gestures reported here, as well as BMI signals that might degrade over time.

As opposed to low-autonomy, task-independent approaches to BMI-driven manipulation that provide generalized capabilities for low-level behaviors such as grasping, reaching and transporting objects (Katyal et al., 2013; McMullen et al., 2014, 2020; Downey et al., 2016), this work focuses on the higher end of the autonomy spectrum, where the robot is assumed to be capable of nominally accomplishing complex bimanual tasks requiring sequential and simultaneous multi-step actions (including ADLs such as eating and getting dressed) and we want to enable BMI-based task customization. As robotics technology continues to advance, we want to leverage these new capabilities while maintaining human control and customization *via* BMI. The limitation of our current approach to collaborative shared control is that it is task dependent and assumes significant competency on the part of the robot, but the strength is that it enables BMI users to personalize task behavior with minimal effort.

In the current study, the participant was able to use a BMI-based shared controller to successfully maneuver two MPLs to perform a bimanual ADL (Figure 4, Supplementary Movie 1). In our shared control strategy, the BMI commands provided by the participant were augmented by the robot controller to enable self-feeding (Supplementary Movie 1). The robot system took inputs from the neural decoder with global offline decoding accuracy of the neural signal at 67.6% (Figure 3), and online target-reach performance of 85%. These are relatively low decoding accuracies in absolute terms, but proved sufficient to meaningfully customize task performance. While higher offline decoding accuracies could possibly contribute to improved performance during the bimanual self-feeding task, it is important to highlight the value of online performance metrics, such as task completion, when evaluating BMI systems for improving function. These demonstrations highlight the value of using neural decoders within an intelligent system, rather than requiring the user to directly control all movements.

While the participant used signals from both hemispheres of the brain to perform the bimanual task, with each robotic arm being controlled by the contralateral hemisphere, recent research suggests the representations of both ipsilateral and contralateral arm movements within the same hemisphere (Ames and Churchland, 2019; Downey et al., 2020). It is possible to control bimanual limbs using neural signals from the motor cortex in one hemisphere, although control of the ipsilateral limb is not always as proficient as the contralateral limb (Downey et al., 2020). Importantly though, the shared control approach presented here could be leveraged for BMI systems that rely on motor recordings from the same hemisphere.

Despite the unequal number of electrodes contributing to gesture decoding of each hand, we achieved comparable accuracies across the two hands. However, the SUA and MUA yield from the arrays in the motor cortex was comparatively lower than from the arrays in the sensory cortex, as described in our previous publication (Thomas et al., 2020). A higher yield in the motor arrays may have resulted in a higher classification accuracy. In addition, when extracting features to train the decoder, we used multiple training samples corresponding to the different phases of the gesture attempt, including movement initiation, sustained movement attempt, and movement release. This approach allowed us to maximize the number of trials used to train the decoder and to ensure that the decoder does not overfit to any single phase of gesture execution. However, this also resulted in more variance among the training samples. Nevertheless, the classifiers were still able to distinguish between several of the gestures with accuracies ranging between 70 and 85% accuracy.

In our approach, the decoder was trained on attempted execution of gestures rather than imagined execution of gestures, which may have resulted in small muscle activations. Given the participant's injury level, imagined gestures would have required active inhibition to avoid any physical movements. BMI users with a complete spinal cord injury would not need to inhibit movement while attempting movement control. As such, training on attempted gestures ensured that the decoder did not learn or depend on neural activity corresponding to inhibitory movement control, which provided a better sense of the performance that can be expected from our motor control approach.

We believe that the shared control technique reported here could be applied to a wide range of ADLs. However, a number of challenges remain. In this study, the positions of target objects were known to the system. This design choice was made to simplify systems integration and focus on shared BMI control for this initial demonstration. Robot perception algorithms are becoming increasingly performant and available (Billard and Kragic, 2019). Future iterations of this system would ideally dynamically estimate the position and orientation of target objects in the environment (e.g., utensils, food, the participant's mouth) *via* cameras co-registered to the robot's coordinate system to provide obstacle avoidance and visual servoing. Additionally, this system utilized multiple shared control parameters (e.g., motion start/end poses, number of DOFs controlled by the user, BMI input activity filtering), which were manually tuned in this study but would ideally be automatically calibrated to increase robustness and scalability to other tasks. Depending on user preference, the robot's verbal interface announcing available DOFs could also be augmented or replaced with a small display. Finally, the robot needs to have knowledge of how to perform a range of tasks and an ability to perform them safely and reliably under the proper context.

This work demonstrates important progress in neurorobotic systems for improving user independence and functionality. In particular, this shared control approach enables user input over many of the steps in the self-feeding task while simultaneously reducing the required DOF to effective complete the task (Figure 2). Compared to other semi-autonomous control approaches for reducing DOF control in assistive technologies (Katyal et al., 2013; McMullen et al., 2014, 2020; Downey et al., 2016), the shared control approach with the human-machine team offers unique advantages in that it provides the user with additional control over individual DOFs and allows for modulation (i.e., relative positioning along varies DOF) during the task. The reduced DOF in a complex, bimanual task is a critical component for allowing a broad range of BMI users, for whom high DOF control signals may not be possible, to benefit from this approach. In essence, the shared control strategy provides better scalability for a variety of BMI users.

Although preliminary, these results mark a critical step in demonstrating the use of an intelligence-assisted BMI for completing necessary, yet sometimes complex, activities of daily living such as cutting and eating food by providing users with shared control of high-DOF bimanual robotic systems.

## Data Availability Statement

Data supporting the conclusions of this article will be made available upon request by the authors, without undue reservation.

## Ethics Statement

The studies involving human participants were reviewed and approved by Johns Hopkins Institutional Review Board. The patients/participants provided their written informed consent to participate in this study. Written informed consent was obtained from the individual(s) for the publication of any potentially identifiable images or data included in this article.

## Author Contributions

DH, AB, MF, and FT: designed the study. DH, AB, TT, MT, RN, and FT: performed the experiments and analysis. MA, DM, GC, PC, MF, and FT: supervised the study. DH, LO, FT, TT, and BW: prepared figures and the manuscript. All authors contributed to editing and revising the manuscript.

## Funding

This work was developed with internal research funding from the Johns Hopkins University Applied Physics Laboratory and National Institutes of Health grant 1R01NS088606. Previous funding from the Defense Advanced Research Projects Agency (DARPA) Revolutionizing Prosthetics program (N66001-10-C-4056) supported implantation surgery, development of the MPLs and of the neural decoding architecture.

## Conflict of Interest

DH is inventor on intellectual property pertaining to robotic manipulation. The remaining authors declare that the research was conducted in the absence of any commercial or financial relationships that could be construed as a potential conflict of interest.

## Publisher's Note

All claims expressed in this article are solely those of the authors and do not necessarily represent those of their affiliated organizations, or those of the publisher, the editors and the reviewers. Any product that may be evaluated in this article, or claim that may be made by its manufacturer, is not guaranteed or endorsed by the publisher.
